# Participatory Research as One Piece of the Puzzle: A Systematic Review of Consumer Involvement in Design of Technology-Based Youth Mental Health and Well-Being Interventions

**DOI:** 10.2196/humanfactors.4361

**Published:** 2015-07-09

**Authors:** Simone Kate Orlowski, Sharon Lawn, Anthony Venning, Megan Winsall, Gabrielle M Jones, Kaisha Wyld, Raechel A Damarell, Gaston Antezana, Geoffrey Schrader, David Smith, Philippa Collin, Niranjan Bidargaddi

**Affiliations:** ^1^ Flinders Human Behaviour & Health Research Unit Department of Psychiatry Flinders University Bedford Park Australia; ^2^ Young and Well Cooperative Research Centre Abbotsford, Victoria Australia; ^3^ Mental Health Informatics Research Unit Country Health SA LHN Inc Adelaide Australia; ^4^ School of Medicine University of Adelaide Adelaide Australia; ^5^ Gus Fraenkel Medical Library Flinders University Bedford Park Australia; ^6^ Institute for Culture and Society University of Western Sydney Penrith Australia

**Keywords:** mental health, young people, technology, intervention, participatory, design

## Abstract

**Background:**

Despite the potential of technology-based mental health interventions for young people, limited uptake and/or adherence is a significant challenge. It is thought that involving young people in the development and delivery of services designed for them leads to better engagement. Further research is required to understand the role of participatory approaches in design of technology-based mental health and well-being interventions for youth.

**Objective:**

To investigate consumer involvement processes and associated outcomes from studies using participatory methods in development of technology-based mental health and well-being interventions for youth.

**Methods:**

Fifteen electronic databases, using both resource-specific subject headings and text words, were searched describing 2 broad concepts-participatory research and mental health/illness. Grey literature was accessed via Google Advanced search, and relevant conference Web sites and reference lists were also searched. A first screening of titles/abstracts eliminated irrelevant citations and documents. The remaining citations were screened by a second reviewer. Full text articles were double screened. All projects employing participatory research processes in development and/or design of (ICT/digital) technology-based youth mental health and well-being interventions were included. No date restrictions were applied; English language only. Data on consumer involvement, research and design process, and outcomes were extracted via framework analysis.

**Results:**

A total of 6210 studies were reviewed, 38 full articles retrieved, and 17 included in this study. It was found that consumer participation was predominantly consultative and consumerist in nature and involved design specification and intervention development, and usability/pilot testing. Sustainable participation was difficult to achieve. Projects reported clear dichotomies around designer/researcher and consumer assumptions of effective and acceptable interventions. It was not possible to determine the impact of participatory research on intervention effectiveness due to lack of outcome data. Planning for or having pre-existing implementation sites assisted implementation. The review also revealed a lack of theory-based design and process evaluation.

**Conclusions:**

Consumer consultations helped shape intervention design. However, with little evidence of outcomes and a lack of implementation following piloting, the value of participatory research remains unclear.

## Introduction

### Technology and Youth Mental Health

More than a quarter of young Australians aged 16-24 years old will experience a mental illness in a 12-month period, with anxiety, substance abuse, and mood disorders the most common [1]. Alarmingly, 3 quarters of first episode mental illness occurs before the age of 25 years [2], and it has been reported that only 30% of these younger people are accessing the professional help that would benefit them [1,3]. With that in mind, technology-based mental health resources and interventions, part of Australia’s e-mental health strategy [4], may offer an opportunity to engage the other 70%. The potential of technology, therefore, to increase youth engagement with formal mental health services, particularly in rural and remote contexts where service options can be limited, is yet to be fully realized.

Technology-based mental health care interventions are often cited as methods for providing greater access to and engagement with services [5-7]. A recent review, however, identified only 2 studies that investigated the use of technology to increase engagement with clinical youth mental health services, and a further 3 explored the role of technology as an adjunct to face-to-face therapy [8]. This review detailed promising results and possibilities for the role of technology in creating and augmenting developmentally appropriate and responsive youth mental health services. However, the research included lacked rigor and the dearth of studies highlight the need for more research and development in the field that is guided by an evidence base [8].

Technology-based health interventions commonly suffer from limited uptake and/or adherence [9-13], which may be dependent on methodological issues such as design, particularly how human factors are incorporated [6,12,14]. For example, failing to obtain an in-depth insight into intended consumer behavior and their environments, which is crucial for good design [15]. Guidelines for technology-based mental health design increasingly emphasize the need for formal incorporation of consumer participation into intervention design [6,16-19]. Therefore, engaging young people and their support communities at all stages of development is likely to be crucial in enhancing uptake and adherence of technology-based interventions, particularly those from rural, remote, and disadvantaged communities [20,21].

### Participatory Research

There is a rich history of participatory research with children and young people in the social sciences [22-25]. Participatory research is conducted in partnership *with* the individuals or community of interest and not *on* them, and in this way differs from traditional research. It purports to increase research relevance and usability through improved context appreciation. Other reported benefits of participatory research include greater stakeholder buy-in and improved efficacy and sustainability of research products (or outcomes) [26-29]. When considering the reported average 17-year gap between publication and translation of findings in health care, it is not surprising that participatory methodologies have gained prominence in the field over the last 20 years [5,28,30,31].

Within mental health design research, common participatory methodologies include community-based participatory research (CBPR), participatory action research (PAR), participatory design (PD), and user-centered design (UCD). PAR aims to develop an egalitarian partnership with a chosen community or group to generate positive, self-identified individual-, group-, and community-level change. While the research goals and associated theories of change may vary, PAR and CBPR are different terms for 1 research methodology underpinned by the same core principles. As such, the terms are used interchangeably in the literature depending on the country of origin [32,33]. PD—borne out of British, North American, and Scandinavian traditions—employs iterative design cycles in which knowledge production and research output(s) are shared by researchers and end-users [34]. Unlike PD, UCD is controlled by the design and research professionals, and participation takes on a strictly consultative role; the project is led, and decisions are made, by “experts” [35]. At the other end of the participatory continuum sits consumer-led research (ie, research initiated and/or controlled by consumers), which has recently taken on new life in the context of social media.

Most research has focused on consumer participation in service delivery, with the literature around participation in intervention design via research projects still developing [36]. It is also less common for the intervention development process to be reported [36]. Boote, Telford, and Cooper [37] argue that consumer involvement in research can be rationalized in 2 ways: (1) empowerment—defined as consumer involvement linked to greater autonomy in decision-making for disempowered/marginalized groups; and (2) consumerism—defined as consumer involvement linked to creating outcomes (eg, products, services or interventions) that generate satisfaction and value-for-money, with consumer input directed at improving efficiency, economy, and effectiveness. Each has different implications for the chosen methodology and role of the consumer.

### The Current Review

Given the potential for technology to increase engagement with mental health services, the current review explored the question: “How have participatory methodologies been employed to develop technology-based youth mental health and well-being interventions?”

Youth participation in the development and delivery of mental health services designed to benefit them has received attention and resourcing for some time [38]. On- and offline service-wide youth participation models are well documented and demonstrate a recognition that young people are best placed to judge what works for them given their developmental-specific experience of mental illness [38]. Online services such as Eheadspace [39], beyondblue [40], and ReachOut.com [41] provide examples of youth participation best practice. This review, however, focuses on participatory development of technology-based interventions by research groups, which may include collaboration with services or other health organizations, as compared to youth participation in an existing service. Project teams involved in production and design of technology-based mental health interventions are interdisciplinary and diverse, and their outputs and findings are distributed across multiple channels and fields depending on the discipline focus of the authors. These factors make a review of this kind a complex undertaking. This review has chosen to focus on work titled, indexed, and stored in databases with a mental health focus and, as such, will not have accessed the body of literature that exists in humanities and social sciences databases (particularly around child, youth and consumer rights and youth participation) that are reflective of multiple stakeholder contributions.

Projects that involved consumers in the design and development of interventions spanning the breadth of the mental health intervention spectrum were included to maximize learning opportunities and to gain a broad understanding of participatory processes in this emerging field of research. The aim was to synthesize previous literature and make practical recommendations for mental health technology designers who wish to employ participatory research methods in a youth context. The major concepts under investigation were: (1) the nature of consumer involvement and the participatory process in intervention development; (2) the nature and outcomes of the design process; and (3) the relationship between participatory research and the implementation of research.

By “technology-based” we refer to information and communications technology-based (ICT-based) digital interventions such as health promotion/prevention Web sites, community-focused health promotion/prevention technologies, treatment-focused Web sites/programs/therapies, and other mental health apps, games, and products. The interventions may act as standalone entities or as an adjunct to existing face-to-face treatment or programs. For inclusion in this review, developers need to have adequately defined and documented (ie, via a project report, journal article, conference paper, or thesis) a participatory development/design project.

## Methods

### Search Strategy

A systematic search strategy was used to identify published and unpublished studies that described participatory research mental health projects. Database search strategies employed both resource-specific subject headings (where available) and keywords describing 2 broad concepts—participatory research and mental health/illness (the emphasis on illness terms reflected the focus on treatment-focused interventions). Keywords were often combined using proximity operators in order to increase search sensitivity (generated by SO, RD, SL, and NB). Comprehensive literature searches were undertaken in the following 15 databases: OvidSP Medline (1946-), PubMed, PsycINFO (1806-), CINAHL, Scopus, Web of Science, Informit (health, social sciences, and science and engineering subsets), arXiv.org, ACM Digital Library, and IEEE Xplore Digital Library. Database searches were limited to studies published in English. The time period for searches was database inception to June 2014. Full search strategies for the OvidSP Medline and PsycINFO databases are provided as Appendix 1.

To identify unpublished studies, 3 simplified versions of the search strategy were used in the Google Advanced search engine and results were restricted to PDF documents. Only the first 100 results for each search variant were reviewed for relevance (ie, total n=300). Web sites of relevant conferences were also checked for additional unpublished papers, including: Participatory Design Conference; Special Interest Group on Computer-Human Interaction; and the Computer-Human Interaction Special Interest Group of the Human Factors and Ergonomics Society of Australia. Reference lists of relevant citations were checked and email contact was made with authors to source additional relevant documentation and current information on the intervention. All searches were conducted in June 2014. EndNote X6 (Thomson Reuters) was used to manage all database citations. A first screening of titles/abstracts by a research assistant (MW) eliminated clearly irrelevant citations/documents based on research method and age group. The remaining citations were screened by a second reviewer (SO). Full text articles were sourced when a decision on relevance could not be made by title or abstract alone.

### Inclusion and Exclusion Criteria

All research papers that involved projects judged as having a primary focus on youth mental health and well-being were included in the review, irrespective of whether the mental health focus was related to an existing physical condition. This decision ensured that learnings from the development of interventions spanning the breadth of the health intervention spectrum would inform development of treatment-focused interventions. Specific criteria are outlined below.

Inclusion criteria:

- Mental health or well-being focus (defined in consultation with a multidisciplinary team comprised of clinical mental health, technology and consumer perspectives, and informed by the DSM-V definition of mental disorder) [42]
- English language
- Development and/or design of ICT- or digital technology-based intervention
- Youth-based intervention (or include a youth element)
- Inclusion of participatory research processes or elements thereof

Exclusion criter**i**a:

- Commentaries, opinion pieces, or editorials
- Photovoice studies (judged as a distinct research methodology that does not involve design or development of a technology-based intervention)

### Data Collection and Analyses

A multidimensional framework analysis, adapted from research conducted by Oliver et al [43] and Lorenc et al [44], was employed to categorize research. This involved an iterative approach of familiarization with the literature and gradual development of the conceptual framework based on the broad research question. Concepts were drawn from the literature around participatory research and technology-based health intervention design. The outcome criteria were populated by criteria drawn from previous participatory research evaluation and the information needs of the study [28,37,45,46]. Due to the exploratory nature of the review, all levels of evidence were considered. Refer to Textbox 1 for definitions of concepts used and their relationship to the areas of investigation. Each study was evaluated by 2 members of the research team using the definitions in Textbox 1. Discrepancies were discussed and consensus reached. A third member of the team was consulted if required.

**Framework analysis.**

Background Information

- Participatory methodology—which participatory methodology underpins the research?
- Project context—who developed the project? Who carried it out? Who funded it?
- Nature of intervention and intended consumers—description of intervention and intended end users.

Nature of Consumer Involvement and the Participatory Process

- Rationale for consumer involvement—empowerment (greater autonomy in decision making for disempowered/marginalized groups) or consumerism (satisfaction and value-for-money, consumer used to improve efficiency, economy and effectiveness) [37].
- Mode of consumer participation—*contractual* (people are contracted into the projects of researchers to take part in enquiries or experiments), *consultative* (people are asked for their opinions and consulted by researchers before interventions are made), *collaborative* (researchers and local people work together on projects designed, initiated and managed by researchers), *collegiate* (researchers and local people work together as colleagues with different skills to offer, in a process of mutual learning where people have control) [46]. Taken from agricultural research, Bigg’s [46] modes of participation simplify Arnstein’s ladder of citizen participation [47] and were reproduced in Cornwall and Jewkes’ paper on participatory research [28].
- Representation (of intended users)—referring to spread of representation from affected interests; including how legitimate the representation was seen to be; the diversity of views not just representatives [45].
- Develop a shared vision and goals—who developed the vision and goals for the project? Did end users have a chance to shape the project in any meaningful way? [45].
- Influence on process (opportunities and quality of involvement)—how and where participants participated in the project (ie, at which stages of the process and in what ways) [45].
- Transparency and quality of decision-making—referring to both internal whereby participants understand how decisions are made; and external; whereby observers can audit the process. Can you determine how and why decisions were made in the project? [45]
- Capacity building and learning for participants—have the participants developed relationships, skills and learning that enable them to take part in future processes or projects? [45].
- Accountability and Legitimacy—referring to whether the representative’s core constituencies are satisfied, including expectations. Referring to the outcomes and process are accepted as authoritative and valid (ie, was there any information regarding participant/stakeholder views on participating in the research the research or on the outcome) [45].

Nature and Outcomes of the Design Process

- Theories used to support intervention design—did the author(s) report any specific theories that help guide the intervention development or design?
- Intervention (efficacy)—is there any published work on the efficacy of the intervention?
- Emergent knowledge—referring to the outcome of local knowledge (ie, from end users) on outcome of the research [45].
- Challenges/limitations plus what worked—limitations and strengths of the process

Relationship Between Participatory Research and Implementation

- Champion/leadership—referring to both the internal leadership for the project and champions for the project [45].
- Implementation—was the intended implementation site(s) indicated? Was it integrated into the project?
- Fate of the intervention—was the intervention implemented in practice? (If not, what stage did the project/intervention reach?)

## Results

### Study Selection

**Figure 1:**
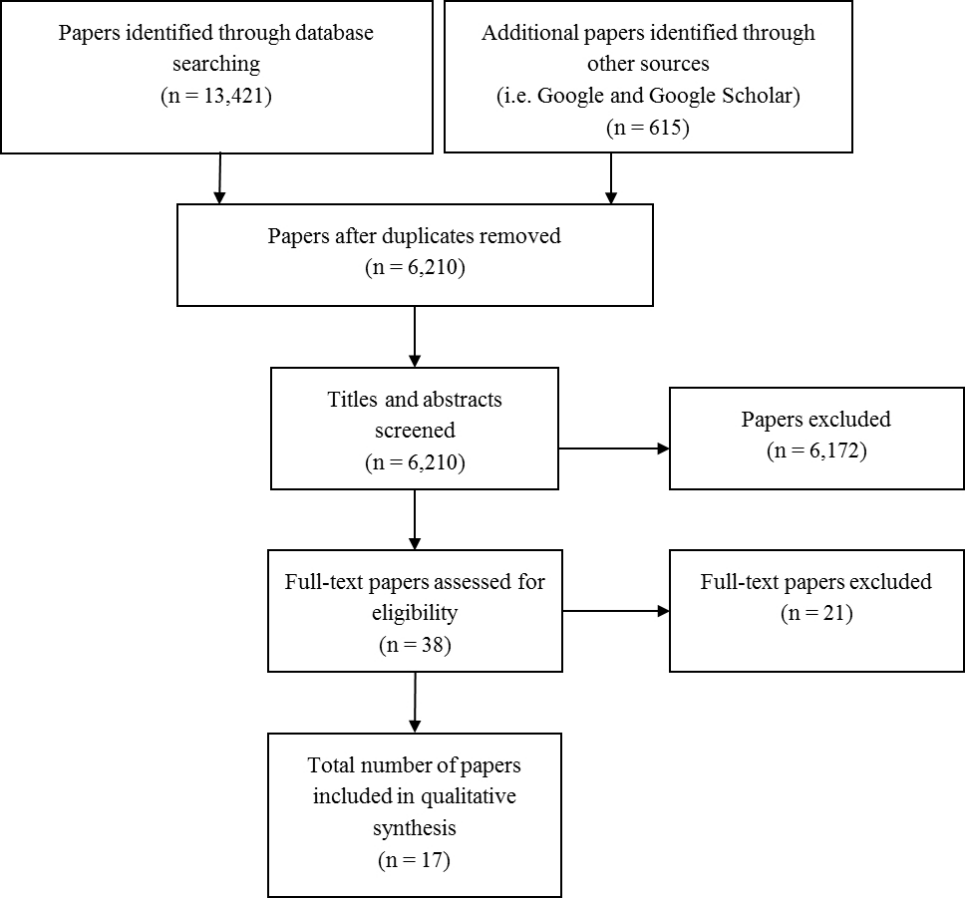
The multiple stages through which studies were selected for inclusion using the PRISMA flow diagram.

In total, 14,021 citations and Web documents were identified through database searches and open Web searching. Once duplicate citations were removed, 6210 items remained for preliminary assessment of relevance. After title, abstract, and full paper screening, 17 studies were chosen for inclusion in this systematic review (Figure 1 and Table 1). Of these, 1 study reached proposal stage [48], and 1 was designed but not developed [49].

**Table 1:** The 17 projects included in the literature review.

Project authors (publication year)	Participatory methodology	Project context	Nature of intervention and intended consumers	Fate of intervention
Carroll, Burge, Robertson, and Rosson (2010) [48]	PAR	Proposed intervention design developed by researchers at Pennsylvania State University.	Preventive Intervention: an on- and offline community network health intervention for university students and families with children with autism.	Not designed or developed (project reached proposal stage).
Coyle and Doherty (2009) [7]	UCD/ collaborative design	Project driven by human computer interaction researchers at Trinity College, Dublin.	Treatment Intervention: 3D computer game (Personal Investigator) to support therapists working with adolescents in public clinical mental health services.	Personal Investigator has undergone initial clinical evaluation over 6 months at multiple sites (n=8 mental health clinicians; and n=22 youth, aged 10-16, gender not reported). Indicated that more formal evaluations of the game were under way, no further information beyond time of publication.
Ekberg, Timpka, and Angbratt, et al (2013) [49]	CPBR with PD process for intervention design	Collaboration between university- and government service-based researchers in Sweden. Grant funded by the Research Council for South-East Sweden.	Preventive Intervention: Online health-promoting community (OHPC) aimed at addressing factors that prevent obesity, including mental health, targeting young people aged 15-20.	Email correspondence with first author indicated a pilot of the OHPC was carried out; however, no formal evaluation was written up. The lead author wished to obtain sustainable funding before launching the OHPC and this is yet to be secured.
Elf, Rystedt, Lundin, and Krevers (2012) [50]	PD	PhD project of first author, in Sweden. Funded by The Swedish Institute for Health Science, the University of Gothenburg, and Vinnvård.	Preventive Intervention: Web-based support system (WBSS) for young caregivers (aged 16-25) living close to someone with mental illness.	During Web site development phase, after previous attempts to pass the Web site on, the original Web site (Molnhopp.nu) was partially redesigned and rebuilt on a different platform (Livlinan.org, Lifeline) run by SPIV (a suicide prevention organization) and a volunteer-run local mental health service for ongoing management. The first author published on the relationship between intended (Molnhopp.nu) and real (Livlinan.org) use of the Web site. Intended and real use were weakly related and dependent on context and the needs/interests of users. The original Web site Molnhopp.nu progressed to a randomized controlled trial (RCT) carried out over 8 months (N=241, aged 16-25 years); WBSS (Molnhopp.nu) n=120 (73% female); folder support (containing information on 24 different kinds of available support services in the community or society) n=121. The intention to treat for the primary outcome (stress) showed no significant differences between the Web group and the folder support group. Stress decreased significantly in the folder group.
Hallett, Brown, Maycock, and Langdon (2007) [51]	PAR	Project driven by a multi-stakeholder participatory action research committee, led by a project officer of the West Australian Aids Council (WAAC) and funded by Healthway (West Australian Health Promotion Foundation).	Preventive Intervention: online, peer-based sexual and mental health promotion (CyberReach) for adult men who have sex with men (MSM) and same sex attracted young people (SSAY). No exact age groups stated, likely to be 14-25 for SSAY and 25+ for MSM.	Stated project objectives met (ie, developing sustainable, transferrable protocols and training, and development of transferrable protocols for peer-based Internet outreach). Paper reports that the piloted intervention became 2 separate services offered by the WAAC: (1) Expanded the existing SSAY to include online outreach and chat; and (2) After a more extensive trial, the MSM service eventually became a national program called “Netreach” offered by the AIDS Councils in Queensland, Victoria, Western Australia, and Tasmania. Netreach primarily provides online chat and support for MSM. Program supported by the Australian Federation of AIDS Organisations and by Gaydar.com.au. No health promotion outcome data available.
Løventoft, Nørregaard, and Frøkjær (2012) [52]	PD with modified form of classic contextual inquiry	University-based research project in Denmark. Project supported by Lundbeck A/S, DIKU, Telenor A/S, HTC Denmark A/S, and PROSA.	Treatment Intervention: mobile phone app aimed at supporting people with depression by assisting with their daily lives. No target age explicitly stated. Youth consumers participating in the study aged 17-24.	Small scale 4-week evaluation of the intervention with participants who assisted with the design process—no further information available on intervention after publication.
Madsen, el Kaliouby, Eckhardt, Hoque, Goodwin, and Picard (2009) [53]	UCD with PD iterative design sessions	Project carried out by MIT Media Lab. Close links with Groden Center and Things That Think Consortium. Funded by National Science Foundation grant (hardware and software prototypes provided by Google and Samsung).	Treatment Intervention: prototype interactive socio-emotional toolkit (iSET) to assist adolescents with autism to improve social interactions (recognition, understanding, and expression of both the user’s and others’ facial expressions via software and hardware).	At time of publication, the iSET intervention was still under development, no further information is available beyond this date.
Matthews and Doherty (2011) [54]	UCD	Project driven by Human Computer Interaction researchers at Trinity College, Dublin (funding source and trial partners not stated).	Treatment Intervention: a mobile phone and online symptom tracking tool (Mobile Mood Diary) to assist adolescents with depression.	Clinical pilot (n=3 therapist, n=9 clients, mean age = 13.78, SD= 2.63, n=3 males and females, respectively) and n=1 parent, across a range of issues, including depression, mood disorders, self-harm, and anger management. No further information available on intervention after time of publication.
Mazzone, Read, and Beale (2008) [55]	UCD with PD	PhD study of first author who was the design researcher in a multidisciplinary research team. UK university-based project led by researchers in developmental psychology and computing. Overall project, joint collaboration between a team of psychologists, interaction designers, and developers. Funded by the HEFCE’s Strategic Development Urban Regeneration Fund, devoted to a consortium of universities in the UK, with additional funding from Esmee Fairburn Foundation.	Treatment Intervention: e-learning product to improve teenagers’ emotional intelligence for pupils (aged 12-15 years old) taken out of mainstream schooling due to behavioral issues (participating consumers were recruited from Pupil Referral Units).	Intervention (Uthink) implemented in Flash by a graphic designer. Uthink evaluation: N=84 (youth aged 14-16, n=72 males, n=12 females), no control group. Significant changes in a number of socio-emotional skills, including stress management, adaptability, and the ability to appreciate relationships between environmental cues and emotions. Participants demonstrated experiencing more care and guidance within friendships and less conflict. Reduced delinquent behavior and a desire to be increasingly challenged in school was also demonstrated. Correspondence with project leads indicated that the game is freely available at the Uthink Web site and is currently being used by schools in Lancashire, England, and is recommended by the Lancashire County Council for use in high schools.
Moen and Smørdal (2012) [56]	Action research with PD workshops	University-hospital collaboration in Norway. Funded by Centre for Rare Disorders and the IT department at Oslo University Hospital. Exploratory study.	Preventive Intervention: wiki-like site offering information, strategies, and support for people (and their families) living with anorectal anomaly focused on “living well.” Indicated all ages were being targeted, but email correspondence with first author indicated a significant youth component.	Email correspondence with the first author indicates there is no outcome paper for the intervention due to employment changes for key contributors. Piloting was undertaken but was challenging due to technical and interoperability problems and lack of professional and organizational support.
Monshat, Vella-Brodrick, Burns, and Herrman (2012) [57]	Participatory research	Researcher-led via Orygen Youth Health Research Centre. Funding: K.M. Australian National Health and Medical Research Council (NHMRC) Public Health Postgraduate Scholarship, J.B. Victorian Health Promotion Foundation (VicHealth) Fellowship, and H.H. NHMRC Practitioner Fellowship.	Preventive Intervention: online mindfulness therapy program (mindfulness awareness training and education (MATE)) targeted at young people aged 14-25.	Pilot testing: (n=11 young people, aged 16-24, gender not reported) evaluated the 6-week MATE program. Focus group (n=7) and interview (n=5) data. No further information available.
Lakey (2014) [58]	Participatory research	Project driven and funded by the National Health Service Greater Glasgow and Clyde as part of their strategic direction for Child and Youth Mental Health. The Greater Glasgow & Clyde NHS, Mental Health Foundation, Snook, and Young Scot were commissioned to carry out project in partnership. Outcome of project is to provide a basis for discussion with stakeholders in the board area to translate findings.	Preventive Intervention: Aimed at exploring the potential of the Internet, social media, and mobile technologies in promoting better mental health and well-being for young people. Multiple planned outputs. Produced digital postcards that act as a guide to staying safe and well online for young people aged 15-21.	Project supported the development of youth-generated ideas for digital interventions to promote youth mental health and well-being. Animated GIFs (youth guide) developed but not available to the public yet. The project also developed other health service/resource design briefs. Work officially launched by Health Board on March 28, 2014. Project opened up connections with innovators across the UK who are willing to collaborate and develop it further. Email correspondence with project lead: project is close to gaining confirmation of funding that will allow development and delivery of recommendations from the project’s first phase.
Owens, Farrand, Darvill, Emmens, Hewis, and Aitken (2011) [36]	Participatory research	Collaboration between university and government service researchers and representatives in the UK. Funded by the National Institute for Health Research.	Treatment Intervention: text-messaging intervention to reduce self-harm for all ages.	Exploratory trial in progress at time of publication. No further information available.
Schmidt (2009) [59]	PAR	Source document was author’s master’s thesis. Youth Voices for Change (YVC) project was a subset of a larger research project (Healthy Youth/Healthy Region) that investigated connections between youth well-being and regional prosperity in the Sacramento, California, region in the US. Participating agencies: The Center for Regional Change at the University of California Davis (UC Davis) in collaboration with other project centers in UC Davis and the West Sacramento Youth Resource Coalition (WSYRC), which led the project. Funding from Sierra Health Foundation and The California Endowment.	Preventive Intervention: Google map (containing youth-produced videos and photos relating the built environment and well-being—eg, favorite, challenge, and adjust places in the community) and project Web page (the project produced other outputs but they were not technology-based). The overall aim was to investigate links between the built environment and youth well-being.	Media products presented at the planned youth community event. Qualitative data (interviews and surveys) indicated that the media products created for the event were perceived as successful by both the youth and the attendees (in terms of overall satisfaction, learning about the community, inspiring discussion, understanding people in the community and its diversity). At time of writing, the thesis indicates that the videos (and other project outputs) were being used by youth groups involved in the project, the Sactown Heroes, to promote their ideas and profile within the community (no clear idea how). The current utilization status of the Google Map is unknown as it was transferred from the project Web page (which was discontinued) and placed on a community Web site. The WSYRC is using the output and connections made as a result of the YVC project to develop a sustainability plan for the Sactown Heroes group as other funding comes to an end.
Stewart, Riecken, Scott, Tanaka, and Riecken (2008) [60]	PAR, youth participation model	Collaboration between university-based researchers and Canadian indigenous youth.	Preventive Intervention: Canadian indigenous youth developed artistic educational videos to address self-identified health concerns. For use in the local and other communities (aimed at high school and university students). Key research question: how can creating videos contribute to expanding health literacy?	Student videos presented at planned showcase event at the end of the school term to an audience of peers, friends, family, and community members. No information as to whether the videos have been used in other communities/contexts as planned.
Valaitis, O’Mara, and Bezaire (2007) [61]	PD	Campus-community partnership between researchers at McMaster University and the local government health unit in Ontario, Canada (rural context). Funded by Health Canada’s Drug Strategy Community Initiatives Fund.	Preventive Intervention: rural youth (aged 14-24) developed a Web site aimed at meeting their specific health promotion needs (with moderated peer support) with a broad aim to address problematic alcohol use. The project also aimed to provide an opportunity and skills for local youth at-risk to develop and implement the health promotion Web site.	No peer reviewed papers published for this study. Project report: the Web site was evaluated over 8 months (2006-2007). No outcome data available on ability of Web site to meet identified health promotion needs. The Youth Spark Web site was functional and updated until late 2014, when it was converted to a Facebook page.
Wadley, Lederman, Gleeson, and Alvarez-Jimenez (2013) [62]	PD	Research project that involved collaboration between universities (from human-computer interaction and clinical backgrounds) and a research supportive youth mental health clinic in Australia. Supported by Victorian Government, University of Melbourne, Telstra Foundation, IBES, the Telematics Trust, and the Helen Macpherson Trust.	Treatment Intervention: online therapy involving psycho-education, peer-to-peer social interaction, advice, and moderation from mental health practitioners for young people with psychosis aged 15-25.	Completed a 4-week safety and acceptability trial (n=20 clients, n=3 clinicians, age and gender not reported). Results of pilot testing results secured funding for a 4-year RCT. Email correspondence with first author indicates that the intervention is in the first year of a RCT—no final outcomes available.

### Characteristics of the Included Studies

Of the 17 projects included in the review, included treatment-focused interventions [7,36,52-55,62]. The remaining 10 were preventive interventions [48-51,56-61]. UCD [7,53-55], PD [50,52,61,62], and PAR [48,51,59,60] were the most common methodologies used (4 projects each). PD provided the sub-framework for an iterative design process in a further 4 projects [49,53,55,56]. UCD or PD methodologies tended to scaffold development of treatment-focused interventions. Three projects were based in the US and Australia, respectively, and 2 each in Ireland, Sweden, England, and Canada. The final 3 were based in Denmark, Norway, and Scotland. The age range of youth involved was 10-26 years old; 5 studies did not report age, 9 did not report gender. Besides age, no other socio-demographic variables were reported.

### Nature of Consumer Involvement and the Participatory Process

Most projects (11 of the 17) involved young people (and other relevant stakeholders) for principally consumerist purposes [7,48,49,51-55,57,58,62]; that is, to create usable, effective, and efficient interventions. A further 2 reported elements of both empowerment and consumerism [36,50]. No projects actively involved youth consumers in the project planning stage, with project aims and goals unreflective of their input.

Overall, consumers were involved in a combination of 3 main stages of research: (1) Needs analysis/design specification; (2) Intervention design/prototyping and development; and (3) Usability and pilot testing. Two projects involved consumers in all 3 stages [52,61]. Projects commonly included consumers, who were most often youth and mental health clinicians (rarely family or caregivers), in the needs analysis/design specification stage [7,49,50,52,54,56,58,61,62]. Some projects entered this stage with a predetermined intervention in mind [49,50,52,54,61], while others operated with a looser set of intended outcomes [7,56,58,62]. Four projects involved consumers in the intervention design/prototyping and development stage [36,52,58,61]. In other projects, consumer involvement involved consulting to refine an existing intervention [51,57] or solely usability and pilot testing [53,55]. The community-based projects of Schmidt [59] and Stewart et al [60] developed community health education tools. They involved consumers at all stages of the project besides initial project planning.

Youth participation was variable, both across and within projects. Overall, 70% of projects reported predominantly consultative consumer involvement [7,49-57,62] and the remaining projects were collaborative in nature [36,58-61]. The projects, therefore, sat in the middle of Biggs’ modes of participation [46]. Youth involvement was consultative in 6 of 7 treatment-focused projects [7,52-55,62], and 4 of these projects involved mental health clinicians as part of the research team [7,54,55,62]. Projects that developed treatment-focused interventions generally involved the most limited forms of consumer input. The highest level of youth participation was evident in the prevention-intervention projects, particularly Lakey, Stewart et al, Valaitis et al, and Schmidt [58-61].

Families, caregivers, and intended implementation-site representatives were under-represented in the projects. Of the 16 carried out, 7 projects clearly identified the intended implementation site and included representatives in the design phase [7,49,51,57,58,61,62]. The Stewart et al [60] and Schmidt [59] projects developed community-education focused interventions with local community representatives; however, it was unclear how widely their products were intended for distribution and thus the specific implementation site(s).

Overall, it was difficult to gain insight into consumers’ views on their participation in the projects (process evaluation) and their outputs (evaluation of the intervention). Three projects involved consumer evaluation of their experience of research [59-61]. These evaluations suggested a general trend toward perceived legitimacy and accountability of the research process and its outputs, but they also served to highlight the different expectations regarding process and outcomes between project/research leads and consumers. Other projects reported informal and anecdotal consumer support for the research process [51,55-57]. In some cases, pilot and small-scale clinical evaluation data were reported [7,51,52,54,57,61,62].

In line with the consumerist rationale for most projects, deliberate capacity building and learning for consumers was limited; only 5 projects involved significant opportunities for this [51,58-61]. These involved development of preventive interventions.

Consumer involvement was seen as crucial to intervention design and development in most projects; emergent knowledge was evident in all project outputs and each made explicit reference to value of consumer involvement in intervention development. Projects reported clear dichotomies around designer/researcher assumptions of effective and acceptable interventions and those of the intended consumer. These differences were present in intervention premise and content [50], and mode of delivery and characteristics/components [52,56,62]. Projects reported compromises between the perspectives, which were evident in the designs. Consumer consultations in the needs analysis/design specification stage were used to underpin and inform intervention design [7,49,50,52,54,56,58,61,62]. Consumers also played a role in tailoring and contextualizing interventions [7,53-55].

Eleven of the 15 completed projects reported challenges with consumer recruitment, capacity, commitment, and reliability [7,36,50-52,54,55,58,59,61,62]. Cited reasons included lack of access to the target consumer group, consumer personal circumstances and/or condition-related factors, and the busy lives of youth. All projects aiming to develop treatment-focused mental health interventions found recruitment and ongoing participation of intended youth consumers difficult to achieve; however, youth consumer attrition during intervention design and development was not specific to development of treatment-focused interventions [50,59,61].

### Nature and Outcomes of the Design Process

Three projects used heuristic guidelines to support intervention design [7,54,62]. Monshat et al [57] was guided by constructs of the Technology Acceptance Model (TAM) [64]. Overall, 4 projects reported use of technology frameworks or theory to guide intervention development [7,54,57,62]. Valaitis et al [61] used logic models to support major project decisions, including those specifically related to intervention design, such as the prototyping process, as well as techniques from scenario-based design [63,65]. Ekberg et al [49] employed design rationales and design space analysis, which detail reasons for and justification of design decisions, to guide development of their intervention [66]. Eight of 17 studies utilized PD methodology or principles to guide intervention development [49,50,52,53,55,56,61,62]. Nine projects mentioned the broad theories (including psychological, health, education, group, empowerment, and cultural) on which the intervention or project were based [6,36,48,51,54-56,60,61] (the details of 2 were found in project reports provided by the authors, not in the published articles [51,61]).

A structured design process, with activities able to scaffold consumer input through the design stages, was seen to be effective in a third of completed projects [49,52,55,58,61]. Use of scenario-based design—which included techniques such as storyboarding, personas [63,65], think-aloud techniques [67,68], and varied methods for capturing user experience and knowledge—was seen to assist the design process. Inspiration/idea progression and prototyping was facilitated by appropriate planning and resourcing with respect to design activities and the space in which they were conducted.

Project flexibility and responsiveness, including the ability to adapt to changing resources, priorities, work styles/preferences, output standards, and deadlines, was often built into design and was a common thread throughout projects that reported high levels of consumer involvement and influence [36,51,59,61]. Projects led by nontechnical researchers also reported the need for integration of technical expertise at all stages of intervention design and development [36,49,61]. A professional appearance of the final intervention product was also seen as important by youth consumers in a number of projects [49,50,62].

In addition, balancing consumer requirements with what was possible technically, ethically, and practically (ie, time and resource, both financial and human, restrictions) was highlighted in 3 projects [49,50,56]. Of particular concern were social and consumer self-authoring components of interventions, privacy, confidentiality, clinical risk, and authenticity of information. Formal outcome data was available for 2 projects [50,55].

### Relationship Between Participatory Research and Implementation

While leadership was not always clearly defined, most projects were researcher-led. Interdisciplinary project teams were common, including researchers or professionals with various combinations of mental health and technology domain expertise. Often, however, 1 discipline had overall responsibility for the project.

Five projects [36,51,58,59,61] reported existing relationships with outside champions who were linked to implementation sites or organizations capable of progressing the project beyond the intervention development stage. In 2 projects, Hallett et al [51] and Valaitis et al [61], project and governance plans were designed such that implementation of the intervention was integrated and a further 4 studies reported established links with intended intervention sites [55,57,58,62]. Stewart et al [60] and Schmidt [59] integrated community-based dissemination of outputs into their project plans. Many projects were, however, exploratory and involved development of technology-based interventions with a limited evidence base.

With the information available at the time of writing, 5 projects had extended beyond the intervention design, development, and pilot stage [50,51,55,61,62]. It is unclear the extent to which outputs from the 2 community-based projects [59,60] were used in a health promotion or prevention capacity beyond the life of the project.

Eleven projects utilized existing relationships and networks to assist with recruitment of target consumers [36,49-51,53,55-59,62]. The benefits of accessing consumers through existing networks was often noted; in particular, this made a significant difference in recruiting consumers with lived experience of mental illness for studies developing treatment-focused mental health interventions [36,53,55,62].

## Discussion

### Nature of Consumer Involvement and the Participatory Process

A strong history of youth participation in mental health research and service development exists, rooted in the empowerment of young people to address service quality and access issues [38]. In contrast, the projects included in this review generally involved consumers for consumerist intentions and in a consultative capacity. This represents a departure from the traditional empowerment and emancipatory rationales for participatory research demonstrated in a minority of projects in this review [36,50,56,59-61]. These increasingly consumerist underpinnings have implications for why and how consumers are asked to participate in research and the degree of mutual benefit that is possible, desired, and ethical.

Eight of the 17 projects explicitly reported using PD methodology or methods to guide intervention development, and others used PD-related design techniques such as user journeys, personas, and workshops. PD originated in the 1970s from a Scandinavian tradition of empowering workers to exercise control over the role of technology in their workplace [69]. Increasingly, however, the application of PD as a methodology or collection of techniques/methods has moved into design underpinned by consumerist principles that emphasize usability, effectiveness, and acceptability of the product [5,19]. This shift was embodied in several projects in this review [49,52,53,55,58,62]. Participatory methodologies with consumerist underpinnings tend to seek information and understanding through consultation and, thus, support a more passive role of the consumer in the research.

In attempting to assess perceived accountability and the legitimacy of the research process and outputs in the studies reviewed, it became clear that researchers are not in the practice of evaluating and reporting on the consumers’ participation experience. This is not only a missed opportunity for consumers to collect data in order to reflect on and learn from their experience of research, but it represents an invaluable source of data from which other projects wishing to conduct participatory intervention design and development could benefit. Email correspondence with 1 author of the studies reviewed revealed that the intervention did not progress any further from the design stage due to possible consumer dissatisfaction with the design, despite the intervention being designed and developed in collaboration with them. This highlights the need for formal assessment of consumer perceptions of accountability and legitimacy of the intervention. Existing literature notes the value derived by researchers and consumers in building in evaluation/reflection cycles, particularly for promoting the dialogue, critical reflection, and trust that are crucial components of high-quality participatory research [23].

While it has been reported that participatory research can enhance recruitment rates [70,71], this review highlights the consumer access, recruitment, and participation challenges faced by projects aiming to develop mental health and well-being interventions, particularly those with a treatment focus that target involvement of consumers with lived experience of mental illness. Those individuals who identify as struggling with mental illness still face stigma and privacy concerns, which restrict use of common recruitment methods such as advertising [62]. Even projects that reported collaboration with mental health services or access to those with lived experience of mental illness noted ongoing participation difficulties with maintaining consumer participation throughout the intervention design and development process [36,62].

Collaborating with existing groups of young people such as schools and youth groups [49,53,55,58-60] or organizations with a strong track record of engagement and outreach with the target consumers [51,56,57] represented a recruitment starting point for multiple projects. However, they too still reported struggling with ongoing participation difficulties. These recruitment concerns are not surprising considering the move into more consumerist-based projects that tend to be less integrated into communities than traditional participatory research.

Personal capacity, reliability, and attrition of consumers, particularly in the treatment-focused intervention development projects, must also be considered [36,50,52,55,61,62]. Todays’ young people contend with a myriad of demands on their time, and projects included in this review experienced this in the form of participant nonattendance, unreliability, and dropout. This effect may be amplified when the youth consumer is currently living with a mental illness. Consumers may also face financial or transport [62] barriers in attending planned project activities that may be related to their age and/or health status. Broadly speaking, participatory research that involves consumers, particularly those who are members of minority or vulnerable populations, carries with it particular ethical considerations that require careful and sensitive negotiation and practical restrictions [72-75]. This is best exemplified in the Løventoft et al project [52], which reported moving from egalitarian principles of PD to a designer-led user-centered approach due to challenges with consumer engagement, retention, and capacity.

The projects with the most extensive youth consumer participation were those in which young people were involved in design and development of health prevention interventions, as exemplified in Stewart et al [60], Valaitis et al [61], Lakey [58], and Schmidt [59]. This nonclinical consumer group is far easier to access and does not have the same privacy, stigma, and personal capacity concerns facing the clinical youth consumers.

Despite this, many studies reported successful participatory research with youth consumers from a range of backgrounds. Participation is greatly assisted by links to existing consumer groups. Integration into the community of interest, via sustained partnerships between academic and nonacademic partners, is a hallmark of participatory research and has previously been shown to enhance recruitment capacity [70,71]. Beyond this, future research projects would be well advised to plan for attrition; both with respect to an ongoing recruitment source and development of materials that can be provided to consumers for seamless integration into the project whenever they choose to engage or reengage. As borne out in this review, participation can and will fluctuate throughout the project and must be planned for and communicated to consumers [59].

Flexibility and open-mindedness, embodied by a willingness to work with a non-static group of consumers and to renegotiate the time, length, style, and content of planned interactions, was repeatedly noted by the projects included in this review [36,51,55,61]. Owens et al [36] in particular highlights the flexibility required by a project when working in an egalitarian manner with consumers. Their intervention became more complex than planned and required extra time and resources to create. Increased cost in terms of necessary resources, time and expertise associated with participatory research [29], along with the need for flexibility in terms of role division, project structure(s), timeframes, and even communication methods have been noted elsewhere [23].

In working with adolescents with behavioral problems, Mazzone et al [55] recommend small groups and many short activities with simple tasks and objectives. They also endorse building in praise and a sense of ownership when working with all youth consumers (see also Dold et al [73]). A structured design process that scaffolds consumers throughout was also found to be effective [49,52,55,58,61]. Given the probable lack of technical and design knowledge of the average consumer (via techniques like storyboarding, think-aloud techniques, and scenario-based design), scaffolding the design process appears to be an important consideration for researchers.

Planning for and understanding consumer expectations of participation in research, along with their self-perceptions as mental health consumers, matters [73]. Given the limited data available regarding consumer experience of research, building reflection and evaluation into research plans should be a focus for future research projects. Ideally, projects wishing to collaborate with youth mental health consumers require committed, youth-supportive research leadership and a process that is well-resourced and supported. Previous research suggests that projects that are age and developmentally appropriate and incorporate meaningful, individualized, empowering, and capacity-building elements improve consumer output and buy-in [59,73,76], which has obvious implications for improving the current recruitment and participation issues.

Recognizing that issues of power and agency are embedded in participatory research with young people, it is important to achieve best practice [23]. When researchers adopt the mind-set that “young people are creative agents who bring about change” [23], participatory research can represent an important opportunity for young people to be recognized and contribute meaningfully.

### Nature and Outcomes of the Design Process

Most studies indicated that consumer participation was integral to good intervention design and development [7,36,49-51,53-56,58,60-62]. Accessing consumers’ implicit domain knowledge was the cornerstone of producing relevant, accessible, and usable interventions and output, which is consistent with prior reviews of participatory research [71,77].

Consumer involvement was associated with flexibility, responsiveness, human-centeredness, and adaptability in design. For example, in their online adaptation of peer-based health promotion for adult men who have sex with men and same sex attracted young people, Hallett et al [51] engaged peer volunteers to develop and pilot the intervention. This allowed the project to be responsive and to adapt the intervention and its evaluation as needed. The peer volunteers provided important information regarding online etiquette and technical proficiency, and during piloting facilitated access to clients and development of rapport and credibility through use of shared language and cultural understandings.

Consumer collaboration significantly altered Owen et al’s [36] text-based self-harm prevention intervention from the original design brief. Researchers originally planned for a replication study in which generic texts were sent at predetermined, high-risk times; the co-design process resulted in a more flexible and human-centered design involving client self-authored texts accessible on demand. Authors noted that the final form and function of the intervention would not have been possible without consumer input.

Successful outcomes require researchers to balance consumer requirements against those of other stakeholders, such as funders and implementation sites, while managing time, resourcing, and ethical considerations. This difficult task requires careful negotiation along with clear and ongoing communication [36,49,50,55,56].

This is best exemplified by analysis of (1) an exit focus group with youth consumers; and (2) youth consumer-designer/researcher email conversations throughout the Elf et al [50] project. Analysis revealed that, as the project progressed, the mind-set of the researcher/designers changed from exploration of ideas with consumers to concrete production of output. This shift in priorities was attributed to increasing pressure around resources (eg, human, financial, time), and delivering technical components on time became the priority over implementing consumer ideas/suggestions.

### Theory to Support Intervention Design

Consistent with prior literature, limited application of theory to guide technology development was evident [17]. As a result, researchers are not maximizing the potential uptake, efficacy, and impact of their interventions. Three projects [7,54,62] used heuristic guidelines to support technology-based intervention design and development. The guidelines emphasize design for outcomes, with mental health professionals, within a UCD framework [6,18]. Consideration of clinical validity, therapist and client usability, along with intervention acceptability, access, engagement, adaptability, and sustainability are also highlighted. Monshat et al [57] was guided by constructs from TAM [64]. Beyond this, theory or models with the ability to explain consumer interaction with the technology were absent.

While the literature is still developing, the behavioral intervention technology model [17] is an example of a model to guide the conceptual and technical architecture of behavior-changing eHealth and mHealth interventions—where eHealth is defined as “internet or other electronic media to disseminate health related information or services” [78] and mHealth as “medical and public health practice supported by mobile devices, such as mobile phones, patient monitoring devices, PDAs, and other wireless devices” [79]. The model guides researchers through development of clinical and usage aims, choice of technical elements and characteristics, and development of the intended workflow associated with the intervention. It assists in translating intervention aims into intervention elements and characteristics [17].

eHealth participatory design best practice advocates for intended users as co-designers and partners in *all* phases of research, along with intervention evaluation criteria that balances youth relevance, meaning, and engagement with existing evidence [19]. This type of theoretical integration is sorely needed in a field constrained by issues with uptake, adherence, and engagement [9-13,17]. Furthermore, persuasive features that “reinforce, change, or shape attitudes or behaviors or both without using coercion or deception” [80] and consumer motivation have had limited application in participatory technology-based mental health intervention design and, therefore, represents a focus of inquiry for future projects [10,14,81,82].

Planning for uptake and established connections with intervention sites were common to projects that successfully implemented their interventions or secured future funding [51,55,58,61,62]. Few projects reported evidence of inclusion of representatives from intended implementation sites in design and development of their interventions, even when accounting for the exploratory nature of some of the projects. A narrow definition of *consumer* may have led to limited representation of intervention site stakeholders in the intervention design phase.

Researchers need to be designing with an implementation site in mind and integrating influential system and organization level representatives into the process. In the case of treatment-focused interventions, mental health teams exist within larger systems that play an important role in acceptance and adoption of new interventions. Intimate knowledge of, and a strong working relationship with, the implementation sites of interest must be a priority of designer-researchers. Wolbling et al [83] argues that “ground-breaking ideas that arise within an existing organization that are not consistent with their values, routines, and overall strategy will be more difficult, if not impossible, to implement.” This assertion has clear implications for a research team wishing to implement new interventions from the outside. Organizational factors such as workplace ICT culture and policy and availability of resources have shown to be facilitators of uptake of ICT in health care [8]. Whilst Coyle et al [6] and Doherty et al [18] account for individual therapist considerations in their heuristic guidelines, they fail to account for organizational and system level factors that can impact on intervention uptake and impact.

Designing with target consumers is crucial. The most commonly reported barriers to uptake of ICT in health care are design and technology concerns including lack of clinical relevance or impracticality; in addition lack of clinician time and perceived ICT skills are frequently reported barriers. On the flip side, facilitators of ICT uptake include system usefulness and functionality, clinical relevance and ease of use [8,84]. This research indicates a clear role for application of theory to guide design and systematic consideration of human factors.

### Limitations

A limitation of this review was the broad inclusion criteria. This is particularly evident with respect to the Schmidt [59] project, which developed community health education outputs to explore youth conceptions of the relationship between the built environment and well-being. Whether these outputs can be categorized as interventions is debatable given the limited detail reported on the project. Despite the fact that youth participation was identifiable in the Owens et al [36] paper, it did not have an exclusive youth focus. It was chosen for inclusion due to the nature of the project and its value in contributing to the aims of the review. In addition, the screening process may have benefited from involvement of a second reviewer to double screen. Evaluation of consumer representation was deemed too complex and broad to explore fully within this review beyond the description provided in the results table (Table 1). Finally, while every reasonable effort was made to find all relevant citations, the broad terminology used to describe the research in question may have resulted in some studies being overlooked, particularly where participatory processes may have been described in the methods sections of papers and not noted in the keywords, title, or abstract. Furthermore, the broad research field means the publication of some studies may not have been amenable to the titles, search terms, and databases that were used to construct this study and answer the research question. Moreover, participatory approaches are used in service settings but not always evaluated with the findings published and as such this work was not represented in the review. This review highlights the need for more research, evaluation, and publication on the use and outcomes of participatory approaches in the design and delivery of technology-based youth mental health services and interventions. The Young and Well Cooperative Research Centre (CRC) [85] is an initiative that prioritizes this connection and creates the required space for the corresponding evidence base to be built.

Given the nascent stage of this field of research and the corresponding exploratory aims of this review, the broad nature of the search terms and studies included facilitated a wide-ranging description and analysis of participatory design and development of technology-based youth mental health and well-being interventions. This ensured that insights and learnings from the breadth of the mental health intervention spectrum were incorporated. The heterogeneous nature of the projects included, however, prevented the number of specific comparisons that could be made between similar projects and intervention types. We also wish to acknowledge that analysis and results of this review attempted to define and summarize a diverse and often ill-defined research field, and in doing so may have inadvertently oversimplified the practical application of participatory intervention design. Finally, in a rapidly evolving field, the search cutoff date meant that highly relevant recent projects found in conference abstracts were not included in the review.

### Conclusions

The current review found limited evidence that consumer consultations lead to routine uptake of interventions in practice; that is, consumer participation does not act as a default implementation or uptake strategy. Overall, strategies aimed at increasing uptake of technology in health care practice are not well understood or reported. A consumerist rationale, which prioritizes acceptability and usability of the intervention, has characterized most projects in this field. It was clear that consumer involvement shaped intervention design in ways that were reported as beneficial by the designers/researchers. While consumer consultations were associated with flexibility, responsiveness, human-centeredness, and adaptability in design, it was not possible to determine the impact of this on intervention effectiveness due to lack of outcome data. The implications for why and how consumers are asked to participate in this field of research and the degree of mutual benefit that is possible, desired, and ethical requires rigorous examination. Participatory intervention design projects are advised to develop flexible and well-resourced project plans, which integrate theory and implementation within the design and make space for reflection, evaluation, and publication of consumer experience of research.

## Appendix

Multimedia Appendix 1 Systematic review search strategy.

## Abbreviations

ICT: information and communications technology
iSET: interactive socio-emotional toolkit
CBPR: community based participatory research
CRC: Cooperative Research Centre
MSM: men who have sex with men
NHMRC: National Health and Medical Research Council
OHPC: online health-promoting community
PAR: participatory action research
PD: participatory design
RCT: randomized controlled trial
SSAY: same sex attracted young people
TAM: technology acceptance model
UCD: user-centered design
WAAC: West Australian Aids Council
WBSS: Web-based support system
YVC: Youth Voices for Change
