# Supplementary material for: Participatory Research as One Piece of the Puzzle: A Systematic Review of Consumer Involvement in Design of Technology-Based Youth Mental Health and Well-Being Interventions
Source: JMIR Hum Factors. 2015 Jul 9;2(2):e12. doi: 10.2196/humanfactors.4361 (PMC4797690; doi:10.2196/humanfactors.4361)
Supplement: Multimedia Appendix 1 [file humanfactors_v2i2e12_app1.pdf]

Search Strategy 1-19 June 14

Database(s): Ovid MEDLINE(R) In-Process & Other Non-Indexed Citations and Ovid MEDLINE(R) 1946 to Present

Search Strategy:

#	Searches	Results
1	Community-Based Participatory Research/	1992
2	(((Participatory or Participative or Cooperative* or Co-operative* or user-led) adj2 (design* or research or approach* or method* or process* or framework* or tool*)) or Co-design* or Codesign* or Co-research*).mp.	6980
3	1 or 2	6980
4	Mental Health/	21607
5	exp Mental Health Services/	74235
6	Mentally Ill Persons/	4363
7	mental disorders/ or adjustment disorders/ or Anxiety/ or exp anxiety disorders/ or anxiety, separation/ or affective symptoms/ or exp aggression/ or exp dissociative disorders/ or delusions/ or exp "schizophrenia and disorders with psychotic features"/ or schizophrenic language/ or paranoid behavior/ or exp eating disorders/ or exp factitious disorders/ or exp impulse control disorders/ or exp "attention deficit and disruptive behavior disorders"/ or child behavior disorders/ or child reactive disorders/ or exp mood disorders/ or depression/ or neurotic disorders/ or exp personality disorders/ or exp "sexual and gender disorders"/ or exp somatoform disorders/ or psychoses, substance-induced/ or exp Self-Injurious Behavior/ or dangerous behavior/ or exp impulsive behavior/ or depersonalization/ or exp obsessive behavior/	659392
8	Resilience, Psychological/	1501
9	(Mental health* or mental illness* or mental disorder* or mentally ill or behavio?ral health or abnormal psych* or depression or depressive or mood disorder* or personality disorder* or psychiatr* or schizophreni* or bipolar or compulsive* or obsessive* or impulsiv* or eating disorder* or self injur* or self harm* or suicid* or psychotic or abnormal psych* or phobi* or psychos* or resilien* or anxiety or anxious or stress* or well- being or wellbeing or wellness).mp.	1552579
10	4 or 5 or 6 or 7 or 8 or 9	1672973
11	3 and 10	1037
12	limit 11 to (english language and humans)	872

.mp.=title, abstract, original title, subject heading word, keyword heading word
/ = Search on Medical Subject Headings (MeSH terms) only
exp = exploded MeSH term

New citations brought in by re-run and addition of new terms n=227 after internal deduplication. 227 citations taken into separate EndNote library.

Database(s): PsycINFO 1806 to May Week 4 2014

#	Searches	Results
1	(((Participatory or Participative or Cooperative* or Co-operative* or user-led) adj2 (design* or research or approach* or method* or process* or framework* or tool*)) or Co-design* or Codesign* or Co-research*).mp.	5674
2	mental health/ or community mental health/ or exp community mental health services/ or community psychiatry/ or exp mental health personnel/ or exp mental health programs/ or exp mental health services/ or primary mental health prevention/	116039
3	mental health services/	26489
4	"Mental Illness (Attitudes Toward)"/ or Homeless Mentally Ill/ or Psychiatric Patients/ or Mentally Ill Offenders/	33228
5	Mental Disorders/ or adjustment disorders/ or anxiety/ or social anxiety/ or exp anxiety disorders/ or generalized anxiety disorder/ or exp neurosis/ or panic disorder/ or phobias/ or exp affective disorders/ or affective psychosis/ or schizoaffective disorder/ or psychiatric symptoms/ or behavior disorders/ or addiction/ or attempted suicide/ or drug abuse/ or juvenile delinquency/ or self mutilation/ or aggressive behavior/ or antisocial behavior/ or behavior problems/ or conduct disorder/ or oppositional defiant disorder/ or antisocial personality disorder/ or exp dissociative disorders/ or exp personality disorders/ or delusions/ or exp schizophrenia/ or exp psychosis/ or borderline states/ or exp eating disorders/ or exp factitious disorders/ or exp impulse control disorders/ or behavioral disinhibition/ or impulsiveness/ or internet addiction/ or pathological gambling/ or pyromania/ or self control/ or exp attention deficit disorder/ or exp Gender Identity Disorder/ or exp somatoform disorders/ or exp self destructive behavior/ or exp stress/	607394
6	exp Coping Behavior/ or exp "Resilience (Psychological)"/ or exp Psychological Endurance/	43841
7	well being/ or life satisfaction/ or positive psychology/	31708

8	(Mental health* or mental illness* or mental disorder* or mentally ill or behavio?ral health or abnormal psych* or depression or depressive or mood disorder* or personality disorder* or psychiatr* or schizophreni* or bipolar or compulsive* or obsessive* or impulsiv* or eating disorder* or self injur* or self harm* or suicid* or psychotic or abnormal psych* or phobi* or psychos* or resilien* or anxiety or anxious or stress* or well- being or wellbeing or wellness).mp.	999615
9	2 or 3 or 4 or 5 or 6 or 7 or 8	1129889
10	1 and 9	1347
11	limit 10 to english language	1294

New citations brought in by re-run and addition of new terms n=293.

Re-run PubMed search

(Participatory design*[tiab] OR Participatory research[tiab] OR Participatory approach*[tiab] OR Participatory method*[tiab] OR Participatory process*[tiab] OR Participatory framework*[tiab] OR Participatory tool*[tiab] OR Participative design*[tiab] OR Participative research[tiab] OR Participative approach*[tiab] OR Participative method*[tiab] OR Participative process*[tiab] OR Participative framework*[tiab] OR Participative tool*[tiab] OR Cooperative design*[tiab] OR Cooperative research[tiab] OR Cooperative approach*[tiab] OR Cooperative method*[tiab] OR Cooperative process*[tiab] OR Cooperative framework*[tiab] OR Cooperative tool*[tiab] OR Co-operative design*[tiab] OR Co-operative research[tiab] OR Co-operative approach*[tiab] OR Co-operative method*[tiab] OR Co-operative process*[tiab] OR Co-operative framework*[tiab] OR Co-operative tool*[tiab] OR User-led design*[tiab] OR User-led research[tiab] OR User-led approach*[tiab] OR User-led method*[tiab] OR User-led process*[tiab] OR User-led framework*[tiab] OR User-led tool*[tiab] OR Co-design*[tiab] OR Codesign*[tiab] OR Co-research*[tiab] OR Community-Based Participatory Research[mh]) AND ((Mental Health[mh] OR Mental Health Services[mh] OR Mentally Ill Persons[mh] OR mental disorders[mh:noexp] OR adjustment disorders[mh] OR Anxiety[mh:noexp] OR anxiety disorders[mh] OR anxiety, separation[mh] OR affective symptoms[mh] OR aggression[mh] OR dissociative disorders[mh] OR delusions[mh] OR "schizophrenia and disorders with psychotic features"[mh] OR schizophrenic language[mh] OR paranoid behavior[mh] OR eating disorders[mh] OR factitious disorders[mh] OR impulse control disorders[mh] OR "attention deficit and disruptive behavior disorders"[mh] OR child behavior disorders[mh] OR child reactive disorders[mh] OR mood disorders[mh] OR depression[mh] OR neurotic disorders[mh] OR personality disorders[mh] OR "sexual and gender disorders"[mh] OR somatoform disorders[mh] OR psychoses, substance-induced[mh] OR Self-Injurious Behavior[mh] OR dangerous behavior[mh] OR impulsive behavior[mh] OR depersonalization[mh] OR obsessive behavior[mh] OR Resilience, Psychological[mh]) OR ((Mental health*[tiab] OR mental illness*[tiab] OR mental

disorder*[tiab] OR mentally ill[tiab] OR behavioral health[tiab] OR behavioural health[tiab] OR psychosocial[tiab] OR depression[tiab] OR depressive disorder*[tiab] OR mood disorder*[tiab] OR personality disorder*[tiab] or psychiatr*[tiab] OR schizophreni*[tiab] OR bipolar[tiab] OR compulsive*[tiab] OR obsessive*[tiab] OR impulsiv*[tiab] OR eating disorder*[tiab] OR self injur*[tiab] OR self harm*[tiab] or suicid*[tiab] OR psychotic[tiab] OR phobi*[tiab] OR psychos*[tiab] OR abnormal psych*[tiab] OR resilien*[tiab] OR anxiety[tiab] OR anxious[tiab] OR stress*[tiab] OR well being[tiab] OR wellbeing[tiab] OR wellness[tiab]) NOT Medline[sb])) AND English[la]

n=587 (30 more citations added using additional terms)

Scopus

(TITLE-ABS-KEY(((**participatory** OR **participative** OR **cooperative*** OR **"Co-operative*"** OR **"user-led"**) PRE/2 (**design*** OR **research** OR **approach*** OR **method*** OR **process*** OR **framework*** OR **tool***)) OR **"Co-design*"** OR **codesign*** OR **"Co-research*"**) AND SUBJAREA(**mult** OR **medi** OR **nurs** OR **vete** OR **dent** OR **heal** OR **mult** OR **ceng** OR CHEM OR **comp** OR **eart** OR **ener** OR **engi** OR **envi** OR **mate** OR **math** OR **phys** OR **mult** OR **arts** OR **busi** OR **deci** OR **econ** OR **psyc** OR **soci**)) AND ((TITLE-ABS-KEY(**"Mental health*"** OR **"mental illness*"** OR **"mental disorder*"** OR **"mentally ill"** OR **"behavioural health"** OR **"behavioral health"** OR **psychosocial** OR **depression** OR **"depressive disorder*"** OR **"mood disorder*"** OR **"personality disorder*"** OR **psychiatr***) AND SUBJAREA(**mult** OR **medi** OR **nurs** OR **vete** OR **dent** OR **heal** OR **mult** OR **ceng** OR CHEM OR **comp** OR **eart** OR **ener** OR **engi** OR **envi** OR **mate** OR **math** OR **phys** OR **mult** OR **arts** OR **busi** OR **deci** OR **econ** OR **psyc** OR **soci**)) OR (TITLE-ABS-KEY(**schizophreni*** OR **bipolar** OR **compulsive*** OR **obsessive*** OR **impulsiv*** OR **"eating disorder*"** OR **"self injur*"** OR **"self harm*"** OR **suicid*** OR **psychotic** OR **phobi*** OR **psychos*** OR **"abnormal psych*"** OR **anxiety** OR **anxious** OR **stress***) AND SUBJAREA(**mult** OR **medi** OR **nurs** OR **vete** OR **dent** OR **heal** OR **mult** OR **ceng** OR CHEM OR **comp** OR **eart** OR **ener** OR **engi** OR **envi** OR **mate** OR **math** OR **phys** OR **mult** OR **arts** OR **busi** OR **deci** OR **econ** OR **psyc** OR **soci**)) OR (TITLE-ABS-KEY(**resilien*** OR **wellness** OR **wellbeing** OR **"well-being"**) AND SUBJAREA(**mult** OR **medi** OR **nurs** OR **vete** OR **dent** OR **heal** OR **mult** OR **ceng** OR CHEM OR **comp** OR **eart** OR **ener**

OR **engi** OR **envi** OR **mate** OR **math** OR **phys** OR **mult** OR **arts** OR **busi** OR **deci** OR **econ** OR **psyc** OR **soci**))) AND LANGUAGE(**english**)

New literature caught with additional terms n=916

Web of Science

Topic=(((Participatory or Participative or Cooperative* or "Co-operative*" or "user-led") NEAR/2 (design* or research or approach* or method* or process* or framework* or tool*)) or "Co-design*" or Codesign* or "Co-research*") AND Topic=("Mental health*" OR "mental illness*" OR "mental disorder*" OR "mentally ill" OR "behavioral health" OR “behavioural health” OR psychosocial OR depression OR "depressive disorder*" OR "mood disorder*" OR "personality disorder*" OR psychiatr* OR schizophreni* OR bipolar OR compulsive* OR obsessive* OR impulsiv* OR "eating disorder*" OR "self injur*" OR "self harm*" OR suicid* OR psychotic OR phobi* OR psychos* OR “abnormal psych*” OR resilien* OR anxiety OR anxious OR stress* OR “well being” OR wellbeing OR wellness)

N=970

Timespan=All years
Search language=English
Language limit=English

CINAHL

Thursday, June 19, 2014

#	Query	Limiters/Expanders	Results
S15	S12 AND S13	Limiters - English Language<br>Search modes - Boolean/Phrase	821
S14	S12 AND S13	Search modes - Boolean/Phrase	844
S13	((Participatory or Participative or Cooperative* OR "Co-operative*" OR "user-led") W2 (design* OR research OR approach* OR method* OR process* OR framework* OR tool*)) OR "Co-design*" OR codesign* OR "Co-research*"	Search modes - Boolean/Phrase	2,420
S12	S1 OR S2 OR S3 OR S4 OR S5 OR S6 OR S7 OR S8 OR S9 OR S10 OR S11	Search modes - Boolean/Phrase	546,049
S11	(depression or depressive or "mood disorder*" or "personality disorder*" or psychiatr* or schizophreni* or bipolar or compulsive* or obsessive* or impulsiv* or "eating disorder*") OR ("self injur*" or "self harm*" or suicid* or psychotic or phobi* or psychos* or resilien*) OR (anxiety or anxious or stress* or "well-being" or wellbeing or wellness)	Search modes - Boolean/Phrase	393,002
S10	"Mental health*" or "mental illness*" or "mental disorder*" or "mentally ill" or "behavio#ral health" or "abnormal psych*"	Search modes - Boolean/Phrase	81,524
S9	(MH "Mental Disorders") OR (MH "Adjustment Disorders+") OR (MH "Mental Disorders, Chronic") OR (MH "Mental Disorders Diagnosed in Childhood") OR (MH "Attention Deficit Hyperactivity Disorder") OR (MH "Child Behavior Disorders+") OR (MH "Separation Anxiety") OR (MH "Neurotic Disorders+") OR (MH "Organic Mental Disorders+") OR (MH "Personality Disorders+") OR (MH "Pregnancy Complications, Psychiatric+") OR (MH "Psychophysiologic Disorders+") OR (MH	Search modes - Boolean/Phrase	304,402

	"Psychotic Disorders+") OR (MH "Sexual and Gender Disorders+") OR (MH "Substance Use Disorders+") OR (MH "Behavior, Addictive+") OR (MH "Compulsive Behavior") OR (MH "Depersonalization") OR (MH "Eating Disorders+") OR (MH "Self-Injurious Behavior") OR (MH "Social Behavior Disorders+") OR (MH "Stress+") OR (MH "Suicide+") OR (MH "Affective Symptoms") OR (MH "Anxiety+") OR (MH "Depression") OR (MH "Behavioral Symptoms")		
S8	(MH "Attitude to Mental Illness")	Search modes - Boolean/Phrase	2,701
S7	(MH "Psychiatric Patients") OR (MH "Mentally Ill Offenders") OR (MH "Psychiatric Care")	Search modes - Boolean/Phrase	11,283
S6	(MH "Psychiatric Home Care")	Search modes - Boolean/Phrase	186
S5	(MH "Psychiatric Service")	Search modes - Boolean/Phrase	301
S4	(MH "Mental Health Services+")	Search modes - Boolean/Phrase	39,665
S3	(MH "Hardiness")	Search modes - Boolean/Phrase	3,034
S2	(MH "Psychological Well-Being")	Search modes - Boolean/Phrase	7,324
S1	(MH "Mental Health")	Search modes - Boolean/Phrase	11,655

No. additional citations with new terms included: n=83

ProQuest– Subsets included

(((title((Participatory OR Participative OR Cooperative* OR "Co-operative*" OR "user-led") PRE/2 (design* OR research OR approach* OR method* OR process* OR framework* OR tool*))) OR (title("Co-design*" OR codesign* OR "Co-research*"))) AND (title("Mental health*" OR "mental illness*" OR "mental disorder*" OR "mentally ill" OR "behavio*ral health" OR "abnormal psych*" OR depression OR depressive OR "mood disorder*" OR "personality disorder*" OR psychiatry* OR schizophrenia* OR bipolar OR compulsive* OR obsessive* OR impulsive* OR "eating disorder*" OR "self injur*" OR "self harm*" OR suicide* OR psychotic OR phobia* OR psychos* OR resilien* OR anxiety OR anxious OR stress* OR "well- being" OR wellbeing OR wellness))) OR (((AB((Participatory OR Participative OR Cooperative* OR "Co-operative*" OR "user-led") PRE/2 (design* OR research OR approach* OR method* OR process* OR framework* OR tool*))) OR (ab("Co-design*" OR codesign* OR "Co-research*"))) AND (ab("Mental health*" OR "mental illness*" OR "mental disorder*" OR "mentally ill" OR "behavio*ral health" OR "abnormal psych*" OR depression OR depressive OR "mood disorder*" OR "personality disorder*" OR psychiatry* OR schizophrenia* OR bipolar OR compulsive* OR obsessive* OR impulsive* OR "eating disorder*" OR "self injur*" OR "self harm*" OR suicide* OR psychotic OR phobia* OR psychos* OR resilien* OR anxiety OR anxious OR stress* OR "well- being" OR wellbeing OR wellness)))

Retrieved 4148
Of these, n=581 unique

PAIS International (1972 - current)
PILOTS: Published International Literature On Traumatic Stress (1871 - current)
ProQuest Central

- ProQuest Family Health
- ProQuest Health & Medical Complete
- ProQuest Health Management
- ProQuest Nursing & Allied Health Source
- ProQuest Psychology Journals
- ProQuest Research Library
- ProQuest Social Science Journals
- ProQuest Sociology (1985 - current)

Social Services Abstracts (1979 - current)
Sociological Abstracts (1952 - current)

Informit

Health, Social Sciences subsets

(“Mental health*” OR “mental illness*” OR “mental disorder*” OR “mentally ill” OR “behavio*ral health” OR “abnormal psych*” OR depression OR depressive OR “mood disorder*” OR “personality disorder*” OR psychiatr* OR schizophreni* OR bipolar OR compulsive* OR obsessive* OR impulsiv* OR “eating disorder*” OR “self injur*” OR “self harm*” OR suicid* OR psychotic OR phobi* OR psychos* OR resilien* OR anxiety OR anxious OR stress* OR “well-being” OR wellbeing OR wellness) AND (((Participatory OR Participative OR Cooperative* OR “Co-operative*” OR “user-led”) AND (design* OR research OR approach* OR method* OR process* OR framework* OR tool*)) OR “Co-design*” OR Codesign* OR “Co-research*”)

Unique = 711 but lots of internal duplication
After internal deduplication n=?

arXiv.org (Cornell University)

Search interface won’t allow searching on more than two terms at a time. Therefore searched design terms only and successively

Restricted to computer science section. N=22 but not relevant

Showing results 1 through 2 (of 2 total) for (ti:"participatory design" OR abs:"participatory design")

1. arXiv:1006.4474 [pdf, other]
   sTeX+ - a System for Flexible Formalization of Linked Data
   Andrea Kohlhase, Michael Kohlhase, Christoph Lange
   Comments: I-SEMANTICS 2010, September 1-3, 2010, Graz, Austria
   Subjects: Software Engineering (cs.SE); Artificial Intelligence (cs.AI)
2. arXiv:0910.1865 [pdf]
   Towards Participatory Design of Multi-agent Approach to Transport Demands
   Yee Ming Chen, Bo-Yuan Wang
   Comments: "International Journal of Computer Science Issues, IJCSI, Volume 4, Issue 1, pp10-15, September 2009"
   Journal-ref: Y. M. Chen and B. Wang, "Towards Participatory Design of Multi-agent Approach to Transport Demands ", International Journal of

Computer Science Issues, IJCSI, Volume 4, Issue 1, pp10-15, September 2009
Subjects: Multiagent Systems (cs.MA)

No matches were found for your search: (ti:"participative design" OR abs:"participative design")

No matches were found for your search: (ti:"user led design" OR abs:"user led design")

No matches were found for your search: (ti:"Co research*" OR abs:"Co research*")

Showing results 1 through 3 (of 3 total) for (ti:"cooperative design" OR abs:"cooperative design")

1. arXiv:1307.2421 [pdf, ps, other]
   Energy Efficient Coordinated Beamforming for Multi-cell MISO Systems
   Yi Huang, Jie Xu, Ling Qiu
   Comments: 6 pages, 2 figures, to be presented in IEEE GLOBECOM 2013
   Subjects: Information Theory (cs.IT)
2. arXiv:0910.2771 [pdf, ps, other]
   Cooperative Interference Management with MISO Beamforming
   Rui Zhang, Shuguang Cui
   Comments: accepted in IEEE Transactions on Signal Processing, June 2010
   Subjects: Information Theory (cs.IT)
3. arXiv:0905.3934 [pdf, ps, other]
   Cooperative encoding for secrecy in interference channels
   O. Ozan Koyluoglu, Hesham El Gamal
   Comments: Submitted to IEEE Transactions on Information Theory (submitted May 2009 and revised September 2010)
   Subjects: Information Theory (cs.IT)

Showing results 1 through 1 (of 1 total) for (ti:"co operative design" OR abs:"co operative design")

1. arXiv:0711.2486 [pdf]
   An annotation based approach to support design communication
   Onur Hisarciklilar (LGS), Jean-François Boujut (LGS)
   Journal-ref: Dans Proceedings of ICED'07 - International Conference on Engineering Design, Paris : France (2007)
   Subjects: Human-Computer Interaction (cs.HC)

Showing results 1 through 10 (of 10 total) for (ti:Co design* OR abs:Co design*)

1. arXiv:1404.4911 [pdf, other]
   Communication Delay Co-Design in $\mathcal{H}_2$ Distributed Control Using Atomic Norm Minimization

Nikolai Matni
Comments: Submitted to the IEEE Transactions on Control of Network Systems
Subjects: Optimization and Control (math.OC); Systems and Control (cs.SY)

2. arXiv:1403.1317 [pdf]
   Hardware software co-design of the Aho-Corasick algorithm: Scalable for protein identification?
   S.M. Vidanagamachchi, S.D. Dewasurendra, R.G. Ragel
   Journal-ref: Industrial and Information Systems (ICIIS), 2013 8th IEEE International Conference on, pp. 321-325, 17-20 Dec. 2013
   Subjects: Computational Engineering, Finance, and Science (cs.CE)
3. arXiv:1208.5604 [pdf, ps, other]
   Optimal co-design of control, scheduling and routing in multi-hop control networks
   F. Smarra, A. D'Innocenzo, M. D. Di Benedetto
   Comments: 51st IEEE Conference on Decision and Control, 2012. Accepted for publication as regular paper
   Subjects: Optimization and Control (math.OC); Systems and Control (cs.SY)
4. arXiv:1201.3881 [pdf]
   Agent-Based μ-Tools Integrated into a Co-Design Platform
   Alain-Jérôme Fougères
   Comments: 10 pages; IJCSI International Journal of Computer Science Issues, Vol. 7, Issue 1, 2010
   Subjects: Human-Computer Interaction (cs.HC); Distributed, Parallel, and Cluster Computing (cs.DC); Multiagent Systems (cs.MA)
5. arXiv:1101.5779 [pdf, other]
   Co-Designing Multi-Packet Reception, Network Coding, and MAC Using a Simple Predictive Model
   Jason Cloud, Linda Zeger, Muriel Médard
   Comments: 8 Pages, 10 Figures, Submitted to WiOpt 2011
   Subjects: Networking and Internet Architecture (cs.NI)
6. arXiv:1004.3263 [pdf]
   DRMS Co-design by F4MS
   Aissam Berrahou, Mourad Rafi, Mohsine Eleuldj
   Comments: International Journal of Computer Science Issues online at this http URL
   Journal-ref: IJCSI, Volume 7, Issue 2, March 2010
   Subjects: Software Engineering (cs.SE)
7. arXiv:1002.0511 [pdf]
   SOPC Co-Design Platform for UWB Systems in Wireless Sensor Network Context
   Daniela Dragomirescu (LAAS), Aubin Lecointre (LAAS), Robert Plana (LAAS)
   Journal-ref: Third International Conference on Systems ICONS Mexico (2008)
   Subjects: Networking and Internet Architecture (cs.NI)
8. arXiv:0910.3736 [pdf, other]

A Fault-tolerant Structure for Reliable Multi-core Systems Based on Hardware-Software Co-design
Bingbing Xia, Fei Qiao, Huazhong Yang, Hui Wang
Comments: 7 pages, 5 figures
Subjects: Hardware Architecture (cs.AR)

9. arXiv:0807.2282 [pdf]
   Hardware/Software Co-Design for Spike Based Recognition
   Arfan Ghani, Martin McGinnity, Liam Maguire, Jim Harkin
   Comments: 6 pages
   Subjects: Neural and Evolutionary Computing (cs.NE); Artificial Intelligence (cs.AI); Computational Engineering, Finance, and Science (cs.CE)
10. arXiv:cs/0612021 [pdf]
   Multimodality and parallelism in design interaction: co-designers' alignment and coalitions
   Françoise Détienne, Willemien Visser
   Journal-ref: Dans COOP'2006 Volume 137 (2006) 118-131
   Subjects: Human-Computer Interaction (cs.HC)

Showing results 1 through 6 (of 6 total) for (ti:Codesign* OR abs:Codesign*)

1. arXiv:1206.1390 [pdf, other]
   Fault-tolerant linear solvers via selective reliability
   Patrick G. Bridges, Kurt B. Ferreira, Michael A. Heroux, Mark Hoemmen
   Subjects: Numerical Analysis (math.NA); Distributed, Parallel, and Cluster Computing (cs.DC); Performance (cs.PF)
2. arXiv:1004.3263 [pdf]
   DRMS Co-design by F4MS
   Aissam Berrahou, Mourad Rafi, Mohsine Eleuldj
   Comments: International Journal of Computer Science Issues online at this http URL
   Journal-ref: IJCSI, Volume 7, Issue 2, March 2010
   Subjects: Software Engineering (cs.SE)
3. arXiv:1002.1154 [pdf]
   Performance Analysis of Software to Hardware Task Migration in Codesign
   Dorsaf Sebai, Abderrazak Jemai, Imed Bennour
   Comments: International Journal of Computer Science Issues, IJCSI, Vol. 7, Issue 1, No. 1, January 2010, this http URL
   Journal-ref: International Journal of Computer Science Issues, IJCSI, Vol. 7, Issue 1, No. 1, January 2010, http://ijcsi.org/articles/Performance-Analysis-of-Software-to-Hardware-Task-Migration-in-Codesign.php
   Subjects: Performance (cs.PF)
4. arXiv:0806.1385 [pdf]
   Control-Scheduling Codesign: A Perspective on Integrating Control and Computing
   Feng Xia, Youxian Sun
   Comments: 7 pages, 2 figures; A review paper

Journal-ref: Dynamics of Continuous, Discrete and Impulsive Systems - Series B, vol. 13, no. S1, pp. 1352-1358, 2006
Subjects: Other Computer Science (cs.OH)

5. arXiv:0806.0130 [pdf]
Feedback Scheduling of Priority-Driven Control Networks
Feng Xia, Youxian Sun, Yu-Chu Tian
Comments: To appear in Computer Standards and Interfaces; doi:10.1016/j.csi.2008.03.020
Subjects: Networking and Internet Architecture (cs.NI)

6. arXiv:0710.4641 [pdf]
UML 2.0 - Overview and Perspectives in SoC Design
Tim Schattkowsky
Comments: Submitted on behalf of EDAA (this http URL)
Journal-ref: Dans Design, Automation and Test in Europe - DATE'05, Munich : Allemagne (2005)
Subjects: Software Engineering (cs.SE)

ACM (DL) Digital Library

Total n=63

15 had EndNote records manually created for them as they appeared to be related to the topic. (Highlighted in yellow below.) Rest listed here for checking

(Abstract:participatory) and (Abstract:"Mental health*" or Abstract:"mental illness*" or Abstract:"mental disorder*" or Abstract:"mentally ill" or Abstract:"behavioral health" or Abstract:"behavioural health" or Abstract:"abnormal psych*") n=4

(Abstract:participatory) and (Abstract:depression or Abstract:depressive or Abstract:“mood or Abstract:disorder*” or Abstract:“personality or Abstract:disorder*” or Abstract:psychiatr* or Abstract:schizophreni* or Abstract:bipolar or Abstract:compulsive* or Abstract:obsessive* or Abstract:impulsiv* or Abstract:“eating or Abstract:disorder*” or Abstract:“self or Abstract:injur*” or Abstract:“self or Abstract:harm*” or Abstract:suicid* or Abstract:psychotic or Abstract:phobi* or Abstract:psychos* or Abstract:resilien* or Abstract:anxiety or Abstract:anxious or Abstract:stress* or Abstract:“well-being” or Abstract:wellbeing or Abstract:wellness) n=59

Taking part: role-play in the design of therapeutic systems
Mark Matthews, Geri Gay, Gavin Doherty

April 2014 **CHI '14:** Proceedings of the SIGCHI Conference on Human Factors in Computing Systems

1 Participatory design strategies to enhance the creative contribution of children with special

needs
Laura Malinverni, Joan MoraGuiard, Vanesa Padillo, MariaAngeles Mairena, Amaia Hervás, Narcis Pares

June 2014 **IDC '14:** Proceedings of the 2014 conference on Interaction design and children

2
Digital portraits: photo-sharing after domestic violence
Rachel Clarke, Peter Wright, Madeline Balaam, John McCarthy

April 2013 **CHI '13:** Proceedings of the SIGCHI Conference on Human Factors in Computing Systems

3
Proceedings of the 2014 conference on Interaction design and children
Ole Sejer Iversen, Lars Elbæk, Bo Stjerne Thomsen, Panos Markopoulos, Franca Garzotto, Christian Dindler

June 2014 **IDC '14:** Proceedings of the 2014 conference on Interaction design and children

4
Utilizing Self-Expression Template Method in User Interface Design - Three Design Cases
Leena Arhippainen, Minna Pakanen

October 2013 **AcademicMindTrek '13:** Proceedings of International Conference on Making Sense of Converging Media

5
Participatory design workshops with children with cancer: lessons learned
Susanne Lindberg

June 2013 **IDC '13:** Proceedings of the 12th International Conference on Interaction Design and Children

6
Understanding and supporting the use of accommodating technologies by adult learners with reading disabilities
Katherine Deibel

September 2006 **SIGACCESS Accessibility and Computing** , Issue 86

7
Designing a mobile diet diary application with and for older adults with AMD: a case study
Lilit Hakobyan, Jo Lumsden, Dympna O'Sullivan, Hannah Bartlett

September 2013 **BCS-HCI '13:** Proceedings of the 27th International BCS Human Computer Interaction Conference

8
Reflection through design: immigrant women's self-reflection on managing health and wellness
Deana Brown, Victoria Ayo, Rebecca E. Grinter

April 2014 **CHI '14:** Proceedings of the SIGCHI Conference on Human Factors in Computing Systems

9
Participatory design of business models
Jacob Buur

August 2012 **PDC '12: Proceedings of the 12th Participatory Design Conference: Exploratory Papers, Workshop Descriptions, Industry Cases - Volume 2**
, Volume 2

10
Participatory design process for an in-vehicle affect detection and regulation system for various drivers
Myounghoon Jeon, Jason Roberts, Parameshwaran Raman, Jung-Bin Yim, Bruce N. Walker

October 2011 **ASSETS '11:** The proceedings of the 13th international ACM SIGACCESS conference on Computers and accessibility

11
Lifestreams: a modular sense-making toolset for identifying important patterns from everyday life
Cheng-Kang Hsieh, Hongsuda Tangmunarunkit, Faisal Alquaddoomi, John Jenkins, Jinha Kang, Cameron Ketcham, Brent Longstaff, Joshua Selsky, Betta Dawson, Dallas Swendeman, Deborah Estrin, Nithya Ramanathan

November 2013 **SenSys '13:** Proceedings of the 11th ACM Conference on Embedded Networked Sensor Systems

12
FATA: um caminho para soluções interativas individualizadas para reabilitação
Luciana Correia Lima de Faria Borges, Lucia Vilela Leite Filgueiras, Cristiano Maciel

October 2010 **IHC '10:** Proceedings of the IX Symposium on Human Factors in Computing Systems

13
Supporting the design contributions of children with autism spectrum conditions
Christopher Frauenberger, Judith Good, Alyssa Alcorn, Helen Pain

June 2012 **IDC '12:** Proceedings of the 11th International Conference on Interaction Design and Children

14
Living Labs as multi-stakeholder platforms for the egovernance of innovation
Francesco Molinari

September 2011 **ICEGOV '11:** Proceedings of the 5th International Conference on Theory and Practice of Electronic Governance

15
Field evaluation of a collaborative memory aid for persons with amnesia and their family members
Mike Wu, Ronald M. Baecker, Brian Richards

October 2010 **ASSETS '10:** Proceedings of the 12th international ACM SIGACCESS

conference on Computers and accessibility

16 Narrative-based elicitation: orchestrating contributions from experts and children
Joan Mora Guiard, Laura Malinverni, Narcis Pares

April 2014 **CHI EA '14:** CHI '14 Extended Abstracts on Human Factors in Computing Systems

17 Design for well-being in China: lessons learned from exploratory workshops
Pei-Chun Chen, Xiaochun Wang

August 2012 **PDC '12: Proceedings of the 12th Participatory Design Conference: Exploratory Papers, Workshop Descriptions, Industry Cases - Volume 2** , Volume 2

18 Using participatory design methods to engage the uninterested
Mariesa Nicholas, Penny Hagen, Kitty Rahilly, Nathalie Swainston

August 2012 **PDC '12: Proceedings of the 12th Participatory Design Conference: Exploratory Papers, Workshop Descriptions, Industry Cases - Volume 2** , Volume 2

19 Words are not enough: empowering people with aphasia in the design process
Julia Galliers, Stephanie Wilson, Abi Roper, Naomi Cocks, Jane Marshall, Sam Muscroft, Tim Pring

August 2012 **PDC '12: Proceedings of the 12th Participatory Design Conference: Research Papers - Volume 1** , Volume 1

20 Breaking boundaries: learning by ARG within an academic conference presentation
Mela Kocher, P. J. Rusnak, Ken Eklund

May 2010 **Futureplay '10:** Proceedings of the International Academic Conference on the Future of Game Design and Technology

21 Artistic participatory practices as a vehicle for togetherness
Rosanne van Klaveren

August 2012 **PDC '12: Proceedings of the 12th Participatory Design Conference: Exploratory Papers, Workshop Descriptions, Industry Cases - Volume 2** , Volume 2

22 A linguistic analysis of group support systems interactions for uncovering social realities of organizations
Feng-Yang Kuo, Chun-Po Yin

March 2011 **Transactions on Management Information Systems (TMIS)** , Volume 2 Issue 1

23
The participatory design of a sound and image enhanced daily planner for people with aphasia
Karyn Moffatt, Joanna McGrenere, Barbara Purves, Maria Klawe

April 2004 **CHI '04:** Proceedings of the SIGCHI Conference on Human Factors in Computing Systems

24
User-driven design of ontology-based, context-aware and self-learning continuous care applications
Femke Ongenae, Filip De Turck

October 2012 **CNSM '12:** Proceedings of the 8th International Conference on Network and Service Management

25
Making chocolate-covered broccoli: designing a mobile learning game about food for young people with diabetes
Marie Glasemann, Anne Marie Kanstrup, Thomas Ryberg

August 2010 **DIS '10:** Proceedings of the 8th ACM Conference on Designing Interactive Systems

26
Design research methods to understand user needs for an etextile knee sleeve
Ceara Ann Byrne, Claudia B. Rebola, Clint Zeagler

September 2013 **SIGDOC '13:** Proceedings of the 31st ACM international conference on Design of communication

27
Framing Participatory Practices in a Large Corporation
Delia Grenville

August 2012 **PDC '12: Proceedings of the 12th Participatory Design Conference: Exploratory Papers, Workshop Descriptions, Industry Cases - Volume 2**
, Volume 2

28
Motherhood and HCI
Madeline Balaam, Judy Robertson, Geraldine Fitzpatrick, Rebecca Say, Gillian Hayes, Melissa Mazmanian, Belinda Parmar

April 2013 **CHI EA '13:** CHI '13 Extended Abstracts on Human Factors in Computing Systems

29
IDEAS: an interface design experience for the autistic spectrum
Laura Benton, Hilary Johnson, Mark Brosnan, Emma Ashwin, Beate Grawemeyer

May 2011 **CHI EA '11:** CHI '11 Extended Abstracts on Human Factors in Computing Systems

30
Top-level decisions through public deliberation on the internet: evidence from the evolution of Java governance

Michael Kaschesky, Reinhard Riedl

May 2009 **dg.o '09:** Proceedings of the 10th Annual International Conference on Digital Government Research: Social Networks: Making Connections between Citizens, Data and Government

31 Cultural hybridity in participatory design
Samantha Merritt, Erik Stolterman

August 2012 **PDC '12: Proceedings of the 12th Participatory Design Conference: Exploratory Papers, Workshop Descriptions, Industry Cases - Volume 2** , Volume 2

32 Participatory design of an online therapy for youth mental health
Greg Wadley, Reeva Lederman, John Gleeson, Mario Alvarez-Jimenez

November 2013 **OzCHI '13:** Proceedings of the 25th Australian Computer-Human Interaction Conference: Augmentation, Application, Innovation, Collaboration

33 Disclosure-Free GPS Trace Search in Smartphone Networks
Demetrios Zeinalipour-Yazti, Christos Laoudias, Maria I. Andreou, Dimitrios Gunopulos

June 2011 **MDM '11: Proceedings of the 2011 IEEE 12th International Conference on Mobile Data Management - Volume 01** , Volume 01

34 An assistive robotic table for older and post-stroke adults: results from participatory design and evaluation activities with clinical staff
Anthony L. Threatt, Jessica Merino, Keith Evan Green, Ian Walker, Johnell O. Brooks, Stan Healy

April 2014 **CHI '14:** Proceedings of the SIGCHI Conference on Human Factors in Computing Systems

35 Poster abstract: PiMi air community:: getting fresher indoor air by sharing data and know-hows
Yixin Zheng, Linglong Li, Lin Zhang

April 2014 **IPSN '14:** Proceedings of the 13th international symposium on Information processing in sensor networks

36 Connecting children to nature with technology: sowing the seeds for proenvironmental behaviour
Bronwyn J. Cumbo, Jeni Paay, Jesper Kjeldskov, Brent C. Jacobs

June 2014 **IDC '14:** Proceedings of the 2014 conference on Interaction design and children

37 Bodily experience and imagination: designing ritual interactions for participatory live-art

contexts
Lian Loke, George Poonkhin Khut, A. Baki Kocaballi

June 2012 **DIS '12:** Proceedings of the Designing Interactive Systems Conference

38 Supporting self-evaluation in local government via KDD
Hye-Chung Kum, Dean F. Duncan, C. Joy Stewart

May 2008 **dg.o '08:** Proceedings of the 2008 international conference on Digital government research

39 The development of novel eyes-free exercise technologies using participatory design
Kyle Rector

January 2014 **SIGACCESS Accessibility and Computing** , Issue 108

40 Participatory design with older adults: an analysis of creativity in the design of mobile healthcare applications
Jennifer L. Davidson, Carlos Jensen

June 2013 **C&C '13:** Proceedings of the 9th ACM Conference on Creativity & Cognition

41 Participatory design with proxies: developing a desktop-PDA system to support people with aphasia
Jordan L. Boyd-Graber, Sonya S. Nikolova, Karyn A. Moffatt, Kenrick C. Kin, Joshua Y. Lee, Lester W. Mackey, Marilyn M. Tremaine, Maria M. Klawe

April 2006 **CHI '06:** Proceedings of the SIGCHI Conference on Human Factors in Computing Systems

42 Embedding participatory design processes into everyday work activities: the case of video consultation services for paraplegics
Julia Klammer, Fred van den Anker, Monique Janneck

November 2010 **PDC '10:** Proceedings of the 11th Biennial Participatory Design Conference

43 Enabling mobile sensing through a DTN framework
Vasilis Maglogiannis, Giannis Kazdaridis, Donatos Stavropoulos, Thanasis Korakis, Leandros Tassiulas

September 2013 **WiNTECH '13:** Proceedings of the 8th ACM international workshop on Wireless network testbeds, experimental evaluation & characterization

44 Children's computer interaction in schools: a case study for promoting healthy computer use
Marina Louise Ciccarelli, Courtenay-Jane Campbell Harris

April 2013 **CHI EA '13:** CHI '13 Extended Abstracts on Human Factors in Computing Systems

45
LiveCompare: grocery bargain hunting through participatory sensing
Linda Deng, Landon P. Cox

February 2009 **HotMobile '09:** Proceedings of the 10th workshop on Mobile Computing Systems and Applications

46
The weight of space: participatory design research for configuring habitable space for new arrival women in Hong Kong
Jackie Yan-Chi Kwok

July 2004 **PDC 04: Proceedings of the eighth conference on Participatory design: Artful integration: interweaving media, materials and practices - Volume 1**
, Volume 1

47
Towards designing more accessible interactions of older people with digital TV
Susan Ferreira, Sergio Sayago, Ernesto Arroyo, Josep Blat

January 2011 **SIGACCESS Accessibility and Computing** , Issue 99

48
Data management techniques for smartphone networks
Demetrios Zeinalipour-Yazti

June 2011 **MobiDE '11:** Proceedings of the 10th ACM International Workshop on Data Engineering for Wireless and Mobile Access

49
hipDisk: understanding the value of ungainly, embodied, performative, fun
Danielle Wilde

May 2012 **CHI EA '12:** Proceedings of the 2012 ACM annual conference extended abstracts on Human Factors in Computing Systems Extended Abstracts

50
Improvising consciousness
Josephine Anstey, Neil Coletta, Dave Pape, Courtney Hatten, Min Young Kim, Debra Burhans, Devin Wilson

June 2013 **C&C '13:** Proceedings of the 9th ACM Conference on Creativity & Cognition

51 Smartphones to facilitate communication and improve social skills of children with severe autism spectrum disorder: special education teachers as proxies
Gianluca De Leo, Gondy Leroy

June 2008 **IDC '08:** Proceedings of the 7th international conference on Interaction design and children

52 Student researchers, citizen scholars and the trillion word library
Gregory Crane, Bridget Almas, Alison Babeu, Lisa Cerrato, Matthew Harrington, David Bamman, Harry Diakoff

June 2012 **JCDL '12:** Proceedings of the 12th ACM/IEEE-CS joint conference on Digital Libraries

53 Interaction design for citizen engagement and digital government
Scott P. Robertson, Elizabeth A. Buie, Dianne Murray

June 2011 **dg.o '11:** Proceedings of the 12th Annual International Digital Government Research Conference: Digital Government Innovation in Challenging Times

54 Enterprise crowdsourcing solutions for software development and ideation
Ranganathan Jayakanthan, Deepak Sundararajan

September 2011 **UbiCrowd '11:** Proceedings of the 2nd international workshop on Ubiquitous crowdsouring

55 Making Education (Double) Count: Boosting Student Learning via Social and Emotional Learning and New Media Literacy Skills
Laurel Felt

July 2010 **eLearn** , Volume 2010 Issue 7

56 RHYME: musicking for all
Harald Holone, Jo Herstad

July 2012 **ICCHP'12: Proceedings of the 13th international conference on Computers Helping People with Special Needs - Volume Part I** , Volume Part I

57 Diversity for design: a framework for involving neurodiverse children in the technology design process
Laura Benton, Asimina Vasalou, Rilla Khaled, Hilary Johnson, Daniel Gooch

April 2014 **CHI '14:** Proceedings of the SIGCHI Conference on Human Factors in Computing Systems

58 Digital government through social networks: how citizens can aggregate their money and votes to define digital government
Britt Blaser, David Weinberger, Joe Trippi

May 2009 **dg.o '09:** Proceedings of the 10th Annual International Conference on Digital Government Research: Social Networks: Making Connections between Citizens, Data and Government

59 The development of novel eyes-free exercise technologies using participatory design
Kyle Rector

April 2014 **CHI EA '14:** CHI '14 Extended Abstracts on Human Factors in Computing Systems

60 Understanding the challenges and opportunities for richer descriptions of stereotypical behaviors of children with asd: a concept exploration and validation
Fnu Nazneen, Fatima A. Boujarwah, Shone Sadler, Amha Mogus, Gregory D. Abowd, Rosa I. Arriaga

October 2010 **ASSETS '10:** Proceedings of the 12th international ACM SIGACCESS conference on Computers and accessibility

61 A participatory design workshop on accessible apps and games with students with learning differences
Lisa Anthony, Sapna Prasad, Amy Hurst, Ravi Kuber

October 2012 **ASSETS '12:** Proceedings of the 14th international ACM SIGACCESS conference on Computers and accessibility

62 What health topics older adults want to track: a participatory design study
Jennifer L. Davidson, Carlos Jensen

October 2013 **ASSETS '13:** Proceedings of the 15th International ACM SIGACCESS Conference on Computers and Accessibility

63 Probing the potential of non-verbal group communication
Petra Sundström, Tove Jaensson, Kristina Höök, Alina Pommeranz

May 2009 **GROUP '09:** Proceedings of the ACM 2009 international conference on Supporting group work

IEEE Xplore DIGITAL LIBRARY

((Mental* OR “behavioral health” OR “behavioural health” OR abnormal OR depression OR depressive OR “mood disorder” OR “personality disorder” OR psychiatr* OR schizophrenia OR schizophrenic OR bipolar OR compulsive OR obsessive OR impulsive OR “eating disorder” OR “self injury” OR “self harm” OR suicid* OR psychotic OR phobi* OR psychos* OR resilience OR resilient OR anxiety OR anxious OR stress OR “well-being” OR wellbeing OR wellness) AND participatory)

((Mental* OR “behavioral health” OR “behavioural health” OR abnormal OR depression OR depressive OR “mood disorder” OR “personality disorder” OR psychiatr* OR schizophrenia OR schizophrenic OR bipolar OR compulsive OR obsessive OR impulsive OR “eating disorder” OR “self injury” OR “self harm” OR suicid* OR psychotic OR phobi* OR psychos* OR resilience OR resilient OR anxiety OR anxious OR stress OR “well-being” OR wellbeing OR wellness) AND participatory)

n=39
Search is basic as limited to 5 wildcards and can’t truncate within “”. Need quotes for phrase searching. Co-design etc. would not work in addition to participatory.
